# Supplementary material for: Pulmonary Embolism in Pregnancy: A Review for Clinical Practitioners
Source: J Clin Med. 2024 May 13;13(10):2863. doi: 10.3390/jcm13102863 (PMC11121909; doi:10.3390/jcm13102863)
Supplement: Supplementary file 1 [file jcm-13-02863-s001.zip › jcm-2958489-supplementary.pdf]

Supplement Table S1. Radiation doses for 256-Slice CTPA.

	BMI (kg/m^2)			
**Person exposed**	**19.7**	**22.9**	**26.3**	**30.1**
**Maternal effective dose (mSv)**	1.00	2.05	2.24	3.43
**Embryo/fetal dose (mGy)**	0.05	0.12	0.12	0.19

BMI - body mass index; CTPA - computed tomographic pulmonary angiography.

Supplement Table S2.

Section	Take Home Message
Clinical Presentation	✓ PAG score can estimate the prevalence of PE in specific risk groups of pregnant patients.
Laboratory Test Values	✓ D-dimer alone to exclude PE does not provide sufficient negative predictive value in pregnancy. Use d-dimer measurement according to YEARS algorithm.
Electrocardiogram	✓ ECG has low sensitivity as a diagnostic test for PE
Imaging	✓ Chest radiography may rule out non-PE-related diagnoses such as pneumothorax or pneumonia<br>✓ Echocardiography in hemodynamically unstable patients supports the clinical diagnosis<br>✓ CUS should be interpreted with the inclusion of C-PTP<br>✓ CTPA and perfusion scintigraphy are suitable for ruling out PE during pregnancy<br>✓ Non-contrast magnetic resonance angiography (MRA) has shown potential in the diagnostics of PE in pregnant patients. Additional studies are needed.<br>✓ Perfusion scanning produces lower effective doses to the breast and fetus than CTPA<br>✓ Lung ultrasonography LUS may detect peripheral lesions in PE. Currently, it is considered an experimental method.
Therapy	✓ Anticoagulation with LMWH should be monitored with anti-factor Xa (target values: 0.5–0.80 IU/mL)<br>✓ Thrombolytic therapy should only be used in hemodynamically unstable patients (high-risk pulmonary embolism)<br>✓ There are no prospective randomized studies on catheter-guided therapy, surgical thrombectomy, and extracorporeal membrane oxygenation in pregnant women. CDT is intended for high-risk patients in whom thrombolysis or appropriately dosed anticoagulation has either failed or is contraindicated<br>✓ Surgical thrombectomy should be evaluated in cases where anticoagulation is ineffective, or the patient is hemodynamically unstable

PAG score - The Pregnancy-Adapted Geneva score; PE – pulmonary embolism; ECG – electrocardiogram; CUS - compression ultrasonography; C-PTP - clinical pretest probability; CTPA - computed tomographic pulmonary angiography; V/Q - ventilation-perfusion; CDT - Catheter-directed therapy; LUS – lung ultrasonography.
